# The Association Between In Utero Exposure to Painkillers and Trajectories of Hyperactivity and Emotional Problems in Children with Autism Compared with Neurotypical Peers

**DOI:** 10.3390/children11121558

**Published:** 2024-12-23

**Authors:** Ping-I. Lin, Kyi Shinn Khin, James R. John, Adam K. Walker, Yi-Chia Chen, Nawar Nayeem, Erick Messias

**Affiliations:** 1 Discipline of Psychiatry and Mental Health, School of Clinical Medicine, University of New South Wales, Sydney, NSW 2033, Australia; kyi_shinn.khin@student.unsw.edu.au (K.S.K.); james.john@unsw.edu.au (J.R.J.); a.walker@neura.edu.au (A.K.W.); 2 Department of Psychiatry and Behavioral Neuroscience, Saint Louis University, St. Louis, MO 63103, USA; nawar.nayeem@health.slu.edu (N.N.); erick.messias@health.slu.edu (E.M.); 3 Academic Unit of Child and Adolescent Psychiatric Services, South Western Sydney Local Health District, Liverpool, NSW 2170, Australia; 4 Laboratory of ImmunoPsychiatry, Neuroscience Research Australia, Randwick, NSW 2031, Australia; 5 School of Population Health, University of New South Wales, Sydney, NSW 2033, Australia; z5389701@zmail.unsw.edu.au

**Keywords:** autism spectrum disorder, painkiller, in utero exposure, hyperactivity, emotional problems

## Abstract

**Background/Objectives**: In utero exposure to painkillers has raised concerns regarding its potential impact on neurodevelopmental disorders, such as autism spectrum disorder (ASD). This study investigates the association between in utero exposure to painkillers and trajectories of hyperactivity and emotional problems in children with and without ASD, separately. **Methods:** Data were drawn from 5107 participants enrolled in the Longitudinal Study of Australian Children. Emotional and behavioral problems were assessed using the Strengths and Difficulties Questionnaire at ages 4, 6, and 8 years. ASD diagnosis was determined based on parental self-report by age 12. To examine the association between the exposure and the outcomes, mixed linear models were applied to assess the impact of in utero exposure to painkillers on hyperactivity and emotional problems, controlling for sex, time, and other perinatal risk factors. The interaction term between exposure and time was included to evaluate the effect of exposure on the trajectory over time. **Results**: In utero exposure to painkillers did not significantly affect hyperactivity or emotional problem trajectories in children with ASD. However, in non-ASD children, painkiller exposure was associated with worsening emotional problems by age 8, with males being affected to a greater extent than females. Further, emotional problem scores increased over time by gender, reflecting developmental challenges in early childhood. **Conclusions**: These findings indicate that prenatal painkiller exposure is unlikely to be a major determinant of the severity of neurodevelopmental outcomes in autistic children, but its role in neurodevelopmental outcomes among neurotypical children warrants further investigation. Future research should prioritize precise exposure assessments and integrate multi-environment interactions to further elucidate the long-term impacts of prenatal painkiller use.

## 1. Introduction

In utero exposure to pharmaceutical compounds may impact fetal growth and postnatal health outcomes due to the fetus’s sensitivity to environmental factors, such as maternal drug use. Recent advances in pharmacovigilance and developmental biology have highlighted the complex interactions between maternal medication use and fetal development, revealing both immediate and long-term consequences that can extend well into adulthood [1,2]. The spectrum of potential effects ranges from subtle alterations in physiological processes to severe congenital malformations, neurodevelopmental disorders, and increased susceptibility to various diseases later in life [3,4].

Investigating the impact of in utero exposure to painkillers on the heterogeneity in neurodevelopmental disorders (NDDs) is a critical area of research. First, the widespread use of painkillers during pregnancy, including both over-the-counter and prescription medications, raises concerns about potential effects on fetal neurodevelopment [5]. Recent meta-analyses and cohort studies have highlighted acetaminophen as a key contributor to these concerns, with evidence suggesting a dose-dependent relationship with neurodevelopmental outcomes, including increased risks for attention-deficit/hyperactivity disorder (ADHD) and autism spectrum disorder (ASD) [6,7]. Notably, the association between in utero exposure to painkillers, such as acetaminophen, seems to be more pronounced in autistic children characterized by hyperactivity [8] or maladaptive behaviors associated with emotional regulation difficulties [9]. These lines of evidence suggest that painkiller exposure may exert a greater impact on hyperactivity features than other behavioral and emotional features.

However, the manifestation of neurodevelopmental symptoms, such as hyperactivity and emotional regulation difficulties, is characterized by significant heterogeneity across individuals with NDDs [10]. This heterogeneity is observed not only in symptom presentation and severity but also in developmental trajectories, comorbidities, and responses to interventions [11], and the role of in utero painkiller exposure in contributing to this variability remains elusive, posing challenges in clarifying how in utero exposure to painkillers contributes to the pathophysiology inherent to ASD. Furthermore, the impact of environmental exposures on neurodevelopmental outcomes indeed varies significantly across different developmental stages during childhood [12]. As children grow, their behavioral patterns change, leading to different exposure risks. Previous evidence indicates that the impact of prenatal opioid exposure on children’s cognitive performance varies substantially between the ages of 6 months and 6 years [13]. Studies that factor in heterogeneity based on gender are hence needed to clarify the longer-term impact of in utero painkiller exposure on neurodevelopmental outcomes.

While studies suggest a link between in utero painkiller exposure and NDDs, the specific mechanisms underlying this association remain poorly understood. To have a better understanding of the role of in utero exposure to painkillers in NDDs, such as ASD, it is crucial to investigate how this exposure affects emotional and behavioral components, which are core elements of neurodevelopmental trajectories related to long-term outcomes. The goal of the current study, hence, aimed to fill gaps in our understanding of the mechanisms involved in prenatal exposure and its impact on neurodevelopment. By comparing these outcomes in both autistic and neurotypical children, we can gain insights into the neuropsychological processes influenced by such exposure. This comparative approach enables the identification of specific developmental pathways that may mediate the risk of ASD and disentangle the effects of prenatal exposure on broader neurodevelopmental outcomes. Furthermore, focusing on emotional and behavioral markers bridges the gap between biochemical disruptions caused by prenatal exposures and their observable manifestations in childhood, providing a more nuanced understanding of their role in the etiology of ASD.

To achieve the goal of clarifying the role of in utero exposure in neurodevelopmental outcomes in autistic children compared with their neurotypical peers, the research objectives are described as follows:

Objective 1: To investigate the association between in utero exposure to painkillers and the trajectories of hyperactivity and emotional problems in children with autism spectrum disorder (ASD) compared to neurotypical peers, while accounting for time, sex, and perinatal risk factors.

Objective 2: To explore whether the effects of in utero painkiller exposure on hyperactivity and emotional problems differ by sex and developmental stage, providing insights into potential sex-specific and age-dependent vulnerabilities.

## 2. Methods

### 2.1. Study Design

The study employs a longitudinal birth cohort design utilizing data from the Longitudinal Study of Australian Children (LSAC), a biennial survey that tracks developmental outcomes in a representative sample of Australian children over multiple time points [14].

### 2.2. Participants

The data consisting of 5107 children was sourced from the LSAC. The birth cohort enrolled children at infancy (0–1 year) in 2004 as part of a nationally representative sample. The cohort was established with a stratified sampling design using the Australian Medicare database to ensure broad demographic representation across regions, socioeconomic backgrounds, and family structures. Data collection occurs biennially, capturing comprehensive information on children’s physical, emotional, and behavioral development, as well as their family and environmental contexts.

### 2.3. Materials and Measures

Emotional and behavioral problems were assessed using the scores of two subdomains, including the hyperactivity scale and emotional problems scale, from the Strengths and Difficulties Questionnaire (SDQ) scale [15], which is behavioral screening tool used to assess emotional and behavioral challenges in children and adolescents. The SDQ has been found to exhibit good inter-rater reliability, cross-informant reliability, test–retest reliability, temporal stability, specificity, sensitivity, positive predictive values, and negative predictive values [16]. It comprises 25 items that are grouped into five key subscales: emotional problems, conduct problems, hyperactivity/inattention, peer relationship problems, and prosocial behavior. Each item is rated on a 3-point scale (not true, somewhat true, certainly true), and the scores from the first four subscales contribute to a total difficulties score. The SDQ can be completed by parents, teachers, or the children themselves (for older age groups), providing flexibility for various settings. Its widespread use and strong validation make it an essential tool for identifying children at risk of mental health difficulties, monitoring behavioral development, and evaluating the impact of interventions. We focused on the SDQ data collected from ages 4 to 8 years.

The diagnosis of ASD was based on parental self-report information confirmed by clinicians when the child reached 12 years of age. The presence of in utero exposure to painkillers including acetaminophen, codeine, and aspirin—the primary environmental factor—was based on the maternal self-report responses extracted from the survey section titled “parental health” for the birth cohort, where women were asked about their medication use during the pregnancy.

To consider perinatal risk factors associated with neurodevelopmental outcomes in the current study, we selected two variables based on parental self-reports. One variable is maternal smoking during the pregnancy coded as a binary variable, which has been shown to correlate with hyperactivity or conduct problems of children [17,18,19,20]. Another variable is birth weight coded as a continuous variable, which was selected as a confounder due to its well documented direct or indirect association with hyperactivity [21,22,23,24,25,26,27] and emotion dysregulation symptoms [28,29]. Therefore, these two perinatal factors were treated as confounders to clarify the association between in utero painkiller exposure and the two neurodevelopmental outcomes in the current study. The data on these two variables were collected using parental reports within 12 months from the birth of the participant as part of the first wave of LSAC data so that the recall bias could be ameliorated.

### 2.4. Statistical Analysis

To evaluate whether the association between in utero exposure to painkillers and the two primary outcomes is unique in children with ASD while taking gender and other prenatal environmental risk factors into account, we used a mixed linear model with repetitive measures to examine whether the trajectory of the hyperactivity score was associated with the interaction term consisting of time (ages of 4, 6, and 8 years) and in utero exposure to painkillers while adjusting for time, in utero exposure, gender, and other covariates related to perinatal risk factors for neurodevelopmental outcomes, such as maternal self-reported perceived stress during pregnancy, maternal smoking during pregnancy, and number of gestational weeks, in this birth cohort. Similarly, we used the same statistical model to evaluate whether in utero exposure to painkillers is associated with the trajectory of the emotional problem score. The trajectory of each of the two outcomes was assessed based on measures at three time points: ages of 4, 6, and 8 years. Shapiro–Wilk tests were used to evaluate the distribution of each of these two continuous outcomes. Rank-based inverse transformation was applied to normalize the outcome variable to mitigate inflated type I errors if a significant deviation from the normality assumption was detected (*p* < 0.01) [30]. Wald tests were used to evaluate whether removing any independent variable with a *p*-value ≥ 0.3 from the model would substantially reduce the fit of that model. The analyses were conducted using the Stata Statistical Software: Release 18 (College Station, TX, USA: StataCorp LLC).

## 3. Results

A total of 2355 children (45.8%) experienced in utero exposure to painkillers, and 145 children (2.8%) were found to have a diagnosis of ASD by the age of 12. Table 1 shows the results of the descriptive bivariate analysis that compared exposed versus unexposed children with regards to the key variables. The results indicate that the exposed group, compared with the non-exposed group, has a significantly higher rate of maternal smoking during the pregnancy (15.73% versus 12.76%, *p* < 0.0001) and a lower child’s birth weight (3424.8 gm versus 3397.9 gm, *p* = 0.0036).

Table 2 summarizes the results from the analysis based on mixed linear models, which aimed to assess the effects of in utero painkiller exposure on hyperactivity and emotional problems in children with and without ASD, focusing on interactions with time, sex, and other covariates. Among children diagnosed with ASD, neither painkiller exposure nor its interactions with time or sex significantly impacted hyperactivity or emotional problem scores (e.g., time × painkiller interaction: hyperactivity at age 6: −0.20, *p* = 0.594; emotional problems at age 6: 0.04, *p* = 0.957). Smoking during pregnancy was significantly associated with increased emotional problem scores (coefficient = 0.56, *p* = 0.004), but no age-specific findings related to painkiller exposure reached statistical significance in this group. In non-ASD children, painkiller exposure was marginally associated with lower hyperactivity scores (coefficient = −0.06, *p* = 0.081) but significantly interacted with time and sex in relation to emotional problems. Specifically, painkiller exposure was associated with worsening emotional problems at age 8 (time × painkiller interaction: coefficient = 0.12, *p* = 0.013). Additionally, a significant interaction between painkiller exposure and sex indicates that males experienced a greater severity of emotional problems compared to females across the three time points (painkiller × sex interaction: coefficient = 0.10, *p* = 0.008). Males consistently exhibited worse hyperactivity (main effect: 0.18, *p* < 0.0001) and less emotional problems (coefficient = −0.16, *p* < 0.0001) compared to females. Age effects were evident in emotional problems, with scores worsening as the child aged from 4 to 8 years among non-ASD children (time at age 8: coefficient = 0.16, *p* < 0.0001).

**Figure 1:**
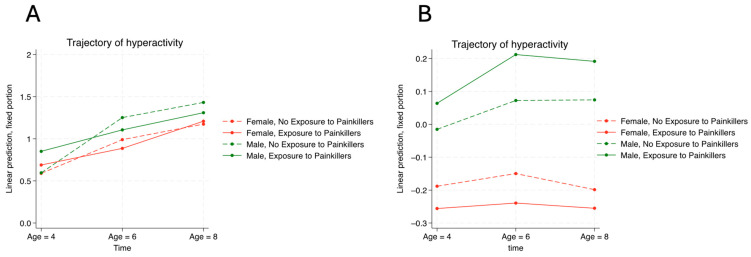
The association between in utero exposure to painkillers and hyperactivity in autistic children (**A**) compared with neurotypical children (**B**).

**Figure 2:**
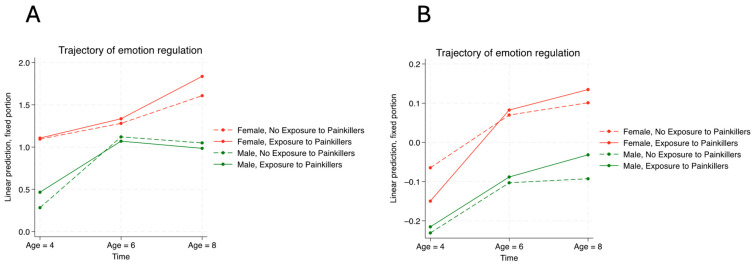
The association between in utero exposure to painkillers and emotional problems in autistic children (**A**) compared with neurotypical children (**B**).

The trajectories of hyperactivity and emotional problems from the ages of 4 to 6 stratified by the presence of ASD diagnosis and in utero painkiller exposure are illustrated in Figure 1 and Figure 2, respectively. Figure 1A shows how the four trajectories were similar to each other, indicating that neither the presence of ASD diagnosis nor the history of in utero painkiller exposure correlated with the trajectory of hyperactivity symptoms from the ages of 4 to 8 for children with ASD. Figure 1B shows that males had more severe hyperactivity symptoms than females for neurotypical children over time, while the relationship between painkiller exposure and hyperactivity exhibited different patterns between males and females. Figure 2A shows that females had worse emotional problems than males among children with ASD, while the severity of emotional problems did not vary between two gender groups. Conversely, Figure 2B shows that females had more severe emotional problems than males among neurotypical children, who also exhibited increasingly severe emotional problems associated with in utero painkiller exposure over time.

## 4. Discussion

### 4.1. Key Findings

The current study has cast novel insights into the role of in utero painkiller exposure in hyperactivity and emotional problems during the early childhood. Overall, no significant associations were observed between in utero painkiller exposure and hyperactivity or emotional problems in ASD children. On the contrary, in utero painkiller exposure could play a role in the trajectory of emotional problems and exhibit a possible sex-dependent effect on hyperactivity symptoms among neurotypical children (see Table 3 for the summary).

### 4.2. Comparison of Current Findings and the Literature

Our findings suggest the lack of a significant association between in utero painkiller exposure and these two neurodevelopmental outcomes, which contrasts with some previous studies that have suggested a link between prenatal painkiller use and neurodevelopmental outcomes, particularly in the context of in utero exposure to opioid analgesics [31,32,33,34]. Nevertheless, our data are in line with a recent study showing limited effects of prenatal exposure to opioid analgesics on the cognitive performances of children in Norway [35]. Notably, the current findings indicate that the use of painkillers during pregnancy may have a stronger association with adverse neurodevelopmental trajectories in neurotypical children compared to children with ASD. These results suggest that the impact of in utero exposure to painkillers on emotional regulation and hyperactivity trajectories may be shaped by neurobiological processes inherent to ASD. Therefore, prenatal painkiller exposure may not be a primary driver of the severity of neurodevelopmental outcomes in autistic children.

Interestingly, our results show that smoking during pregnancy was significantly associated with increased emotional problem scores in children with ASD. This finding supports the existing literature on the detrimental effects of prenatal smoking on neurodevelopment and emphasizes the importance of considering multiple prenatal exposures in assessing developmental outcomes [6,36]. In non-ASD children, our results revealed more nuanced effects of painkiller exposure. The lack of a significant association between painkiller exposure and hyperactivity scores, which is in line with some previous evidence based on clinical and preclinical studies [37,38], still warrants further investigation as it contrasts the conclusions from a meta-analysis based on 7 cohort studies reporting an increased risk of ADHD symptoms following prenatal painkiller exposure [39]. The discrepancy might be due to differences in study populations, types of painkillers considered, or the timing of exposure during pregnancy [37].

For neurotypical children, the worsening of emotional problems at age 8 was associated with painkiller exposure, especially in males. Similarly, the effect of in utero painkiller exposure on hyperactivity symptoms appeared to be more pronounced in males, aligning with the previous evidence suggesting sex-specific effects of prenatal exposures on neurodevelopmental outcomes [40,41]. This finding underscores the importance of considering sex as a biological variable in studies of prenatal exposures and neurodevelopment. The observed age-related changes in emotional problems, with scores worsening non-linearly between ages 4 and 8 among neurotypical children, are noteworthy. These findings likely reflect the developmental challenges children face during the transition from preschool to school age, as they adapt to new social and academic demands [42,43]. Further, our findings on gender differences in emotional and behavioral problems during early childhood are consistent with previous evidence [44,45].

### 4.3. Limitations

Although our study has several strengths stemming from its longitudinal design, it has several limitations that should be considered when interpreting its findings. The survey captured a limited sample size of individuals with ASD, potentially leading to an underpowered comparison, as reflected by the observed lack of association between perinatal risk factors and ASD risk [46]. The reliance on maternal self-reports for in utero painkiller exposure introduces the potential for recall bias, as mothers may inaccurately report the type, dosage, or frequency of use, leading to potential misclassification. Furthermore, the grouping of multiple painkiller types (e.g., acetaminophen, codeine, and aspirin) into a single exposure category limits the ability to isolate the specific effects of individual compounds, which have distinct pharmacological profiles and impacts on fetal development. Additionally, the study does not adequately account for maternal pain conditions that may necessitate painkiller use; these conditions are often associated with increased inflammatory cytokines, which themselves may influence neurodevelopmental outcomes [47], making it difficult to disentangle the effects of painkillers from those of the underlying maternal health conditions. The lack of direct biomarker measurements, such as cord blood or amniotic fluid analyses, further reduces the precision of the exposure assessment, while the absence of information on the timing of painkiller use relative to critical developmental windows limits the ability to understand time-dependent effects. Despite adjusting for key perinatal risk factors, such as maternal smoking and birth weight, residual confounding from other unmeasured factors (e.g., maternal diet, additional medication use, or environmental exposures) may still influence the findings. Lastly, the study’s generalizability is limited to the population of the Longitudinal Study of Australian Children, which may not reflect outcomes in populations with different environmental, genetic, or healthcare factors. These limitations underscore the need for future studies with more precise exposure measurements, biomarker validation, and detailed consideration of maternal health and environmental variables.

### 4.4. Conclusions and Future Research Direction

The current findings highlight the dynamic and evolving nature of emotional development in children. They underscore the significant, long-term impact that prenatal exposures can have on emotional regulation and emphasize how these early-life factors can influence the trajectory of mental health outcomes over time. This has critical implications for both clinical practice and public health strategies, particularly in the prevention and early intervention of mental health conditions relevant to in utero painkiller exposure. As the effects of prenatal exposures on emotional regulation are often subtle and occur over extended periods, it becomes essential for future research to incorporate longitudinal, prospective study designs that can better capture the timing and progression of such impacts relative to key developmental windows.

From a clinical perspective, these findings could lead to more personalized, early interventions targeting emotional regulation and other neurodevelopmental risks associated with ASD. By recognizing the impact of prenatal exposures, clinicians could develop preventative strategies for at-risk populations, focusing on early identification and intervention during crucial developmental milestones. Public health interventions could also benefit from a more nuanced understanding of environmental and genetic factors that contribute to ASD and emotional dysregulation, enabling more effective targeting of public health resources and outreach.

Future studies investigating ASD risk factors should adopt a developmental psychopathology framework, incorporating prospective designs with precise measurement and timing of exposures relative to critical developmental periods. Additionally, these studies should account for the dynamic gene–environment interplay by employing genetically informed methodologies [48].

## Figures and Tables

**Table 1:** Characteristics of the children with in utero exposure to painkillers versus peers without in utero exposure to painkillers (N = 2355).

	Exposure (+)		Exposure (−)		
	Percentage	SD	Percentage	SD	*p*-Value
ASD Diagnosis	3.99%	0.30%	3.74%	0.25%	0.492
Gender (Male)	51.09%	0.60%	51.03%	0.55%	0.939
Maternal Smoking During Pregnancy	15.73%	0.44%	12.76%	0.37%	<0.001
	**Mean**	**SD**	**Mean**	**SD**	***p*-Value**
Birth Weight (gm)	3424.79	555.92	3397.89	579.2	0.0036
Hyperactivity (Mean) *	3.45	2.35	3.37	2.3	0.0843
Emotional Problems *	1.69	1.75	1.63	1.74	0.0955

* Measures were based on the average across three time points at the ages of 4, 6, and 8.

**Table 2:** The analysis results based on mixed linear models with repetitive measures.

Children with ASD				
Predictor	Hyperactivity		Emotional Problems	
	Coefficient	*p*-Value	Coefficient	*p*-Value
Time				
Age 6	0.4	0.165	0.19	0.568
Age 8	0.59	0.068	0.51	0.309
Painkiller Exposure	0.11	0.831	0.03	0.960
Painkiller Exposure × Time Interaction				
Painkiller × Time (Age 6)	−0.2	0.594	0.04	0.957
Painkiller × Time (Age 8)	−0.06	0.887	0.22	0.746
Sex (Male versus Female)	0.01	0.983	−0.8	0.118
Painkiller × Sex	0.14	0.819	0.17	0.802
Maternal Smoking	−0.07	0.785	0.56	0.004
Birth Weight	0	0.896	0	0.711
Income Group	−0.04	0.673	0.01	0.943
**Neurotypical Children**				
**Predictor**	**Hyperactivity**		**Emotional Problems**	
	**Coefficient**	***p*-Value**	**Coefficient**	***p*-Value**
Time				
Age 6	0.03	0.301	0.13	<0.001
Age 8	−0.01	0.569	0.16	<0.001
Painkiller Exposure	−0.06	0.081	−0.08	0.116
Painkiller Exposure × Time Interaction				
Painkiller × Time (Age 6)	−0.02	0.761	0.1	0.063
Painkiller × Time (Age 8)	0.01	0.738	0.12	0.013
Sex (Male versus Female)	0.18	0.0001	−0.16	<0.001
Painkiller × Sex	0.14	0.008	0.1	0.115
Maternal Smoking	0.25	0.0001	0.03	0.62
Birth Weight	0	0.17	0	0.135
Income Group	−0.14	0.0001	−0.09	<0.001

**Table 3:** A summary of the key findings regarding the hyperactivity and emotional problems occurring from the ages of 4 to 8.

	Factors Influencing Overall Hyperactivity Symptoms	Factors Influencing Overall Emotional Problems	Does Painkiller Exposure Influence the Trajectory of Hyperactivity Symptoms?	Does Painkiller Exposure Influence the Trajectory of Emotional Problems?
Children with ASD	None	None	No	No
Neurotypical Children	Gender; Maternal Smoking; Family Income	Gender; Gender–Painkiller Exposure Interaction; Family Income	No	Yes

## Data Availability

The datasets presented in this article are not readily available because access to them needs to be approved by the Longitudinal Study of Australian Children. Requests to access the datasets should be directed to the corresponding author.
